# Hepatic Stellate Cell-derived IL-11 Exacerbates Liver Fibrosis via Interplay between HSCs and Macrophages

**DOI:** 10.7150/ijbs.119659

**Published:** 2026-01-01

**Authors:** Yu Zhang, Fangfang He, Mozi Lei, Wenhui Fan, Xingyu Liu, Ying Tao, Weinan Wang, Bingshun Wang, Likun Gong, Jing Chen

**Affiliations:** 1 State Key Laboratory of Drug Research, Shanghai Institute of Materia Medica, Chinese Academy of Sciences, Shanghai, China.; 2 School of Pharmacy, University of Chinese Academy of Sciences, Beijing, China.; 3 School of Chinese Materia Medica, Nanjing University of Chinese Medicine, Nanjing, China.; 4 Department of Biostatistics, Clinical Research Institute, Shanghai Jiao Tong University School of Medicine, Shanghai, China.; 5 China-Serbia "Belt and Road” Joint Laboratory for Natural Products and Drug Discovery, Shanghai Institute of Materia Medica, Chinese Academy of Sciences, Shanghai, China.

**Keywords:** liver fibrosis, IL-11, hepatic stellate cells, macrophages, cell-cell interaction

## Abstract

It has been a consensus that hepatic microenvironment composed by the non-parenchymal cells networks play a critical role during liver fibrogenesis, with the crosstalk between hepatic stellate cells (HSCs) and macrophages being of paramount importance. Interleukin 11(IL-11) has been implicated as a pro-fibrogenic cytokine, where its function in/between hepatocytes and HSCs has been revealed. But whether IL-11 participates in the interaction of HSCs and macrophages and related mechanism remains obscure. Our research demonstrates that HSC-derived IL-11 operates through a dual mechanism of autocrine activation and paracrine reprogramming to drive the fibrosis. AAV6-mediated IL-11 overexpression in the HSCs aggravated hepatic fibrosis induced by CCl_4_ in C57/B6 mice, accompanied by a marked increase of M2 macrophages. Mechanistically, the autocrine signaling of IL-11 activated HSCs directly, potently enhancing the contractility, migration, and collagen production of HSCs through GP130-SFK-YAP pathway. Furthermore, IL-11 also functioned as a paracrine signal of HSCs activation that synergized with IL-4 to polarize macrophages into a profibrotic M2-like phenotype. This reprogramming was achieved through the coordinated activation of PI3K-mTOR signaling to promote TGF-β synthesis and STAT3 pathway to elevate chemokine levels. The necessity of macrophages in this process was proven when their depletion blunted the pro-fibrogenic effects of IL-11 overexpression. Consequently, therapeutic inhibition of IL-11 with a nanobody alleviated fibrosis and reversed macrophage polarization. Our findings proposed a self-amplifying loop where HSC-derived IL-11 directly activates fibrogenesis and simultaneously reprograms macrophages to create a feed-forward cycle that relentlessly drives disease progression.

## Introduction

Liver fibrosis is initially a defensive and self-repairing process to restore and maintain the protective barrier when the liver faces a wide range of pathological injuries. However, the continuous injuries in chronic liver disease (CLD) promote the hyperactivation of fibrotic pathway to impair hepatocyte function and disorganize liver structure 1. Since liver fibrosis acts as ‌the indispensable pathological phenotype driving the evolution of CLD into cirrhosis, anti-liver fibrosis has been recognized as one of the effective strategies for treating CLD. Myofibroblasts are the predominant determinants during organ fibrosis 2. Owing to the emergence and development of single-cell RNA-sequencing technology, multiple types of cells in the liver are identified as the sources of myofibroblasts, like liver sinusoidal endothelial cells (LSECs) 3 and macrophages 4, but hepatic stellate cells (HSCs) are still the main origin of myofibroblasts 5. Activated HSCs by fibrogenic mediators can migrate to the injury site and produce extracellular matrix (ECM) (e.g. collagen type I and III) to form a fibrous scar 6, 7. In this case, it is important to investigate the underlying mechanism of HSCs activation in order to discover potential strategy for anti-liver fibrosis.

During the activation process, HSCs undergo the two stages of “initiation” and “perpetuation” 8. At the initiation stage, HSCs respond to microenvironment stimuli especially fibrogenic and mitogenic factors, and develop an increased phenotype of contractability and fibrogenesis 9. In the subsequent perpetuative phase, the characterization of initially activated HSCs was amplified, which will respond to large quantities of chemokines and growth factors secreted from other adjacent cells including hepatocytes and non-parenchymal cells (NPCs)^10^. Therefore, to explore the crosstalk between NPCs and HSCs could deepen our understanding of HSCs activation during liver fibrosis. Macrophages are the largest population of innate immune cells in the liver, which are classified into monocyte-derived macrophages (MoMFs) and resident Kupffer cells (KCs) according to their origin. Generally, the recruited MoMFs and the polarized KCs by microenvironment stimuli produce large amounts of inflammatory cytokines and chemokines (TNF, CCL2, CCL5) as well as profibrotic and mitogenic cytokines (TGF-β and PDGF), which will directly influence HSCs activation 11. However, there are still many details and the specific roles of key cytokines are controversial or unclear. For example, both MoMFs and KCs can produce TGF-β which can transdifferentiate HSCs into myofibroblasts 12, 13, though some researches hold the view that TGF-β is mainly secreted from MoMFs and KCs-derived TGF-β accounts for phagocytic and anti-inflammatory functions 14, 15.

Interleukin-11(IL-11) belongs to the interleukine-6 (IL-6) family and it enters into the researchers' vision for its function in promoting thrombopoiesis. Nevertheless, recently IL-11 is regarded as a driver for fibrosis among several organs including heart, lung, kidney and liver 16. In the hepatic fibrotic progression, IL-11 intermediates the crosstalk between hepatocytes and HSCs: on one hand, hepatocytes suffered from lipid stimuli could secrete IL-11 which activates HSCs to lead to ECM accumulation, on the other hand, HSCs responding to various stimuli in liver microenvironment could produce IL-11 to promote hepatocytes apoptosis via increasing reactive oxygen species (ROS) 17, 18. Meanwhile, both hepatocytes and HSCs-originated IL-11 have the autocrine effects on themselves by promoting hepatocytes apoptosis and HSCs activation separately. Even though IL-11 is a cytokine, its role on immune microenvironment during the deterioration of liver fibrosis has not been fully elucidated.

In the current work, we investigated the effects and mechanisms of HSCs-originated IL-11 on autocrine activation of HSCs and macrophage polarization, and the crosstalk between HSCs and macrophages, hoping to inspire new insights into how IL-11 regulates the mutual interaction among NPCs in the liver and to assess the value of IL-11-targeting strategies in modulating the immune microenvironment during fibrosis.

## Materials and Methods

### Mice

All animal experiments complied with the Institutional Ethical Guidelines and were approved by the Institutional Animal Care and Use Committee, Shanghai Institute of Materia Medica, Chinese Academy of Sciences (2022-12-RJ-290, 2023-12-GLK-39 and 2024-10-GLK-74). 8-week-old male C57BL/6J mice were purchased from Huafukang Biotechnology Co., Ltd (Beijing, China) and placed in a controlled environment (12h dark/12h light cycle, 60-70% humidity, 23±1°C) with free access to water and normal chow diet.

For CCl_4_-induced liver fibrosis model (2022-12-RJ-290), the mice were received CCl_4_ (10%) dissolved in olive oil by i.p. injection at 5 mL/kg of body weight, three times a week. To examine the effect of IL-11 *in vivo*, 8-week-old mice were administered pAAV6-CBh-3xFLAG-EF1-GdGreen-WPRE (Vector group, n = 4) or pAAV6-CBh-Il11-3xFLAG-EF1-GdGreen-WPRE (IL-11 OE, n = 4) by tail vein injection. Besides, they were injected with CCl_4_ in parallel for 4 weeks as mentioned above. Another program was that 8-week-old mice were administered pAAV6-CBh-3xFLAG-EF1-GdGreen-WPRE (n = 4) or pAAV6-CBh-Il11-3xFLAG-EF1-GdGreen-WPRE (n = 4) by tail vein injection 4 weeks previously, followed by 2 weeks i.p. injection of CCl_4_. All mice were sacrificed to obtain serum and liver samples at 24h post the last injection of CCl_4_.

For macrophage depletion experiment (2024-10-GLK-74), 8-week-old mice were administrated pAAV6-CBh-3xFLAG-EF1-GdGreen-WPRE (n = 3) or pAAV6-CBh-Il11-3xFLAG-EF1-GdGreen-WPRE (n = 3) by tail vein injection. After 4 weeks, they were all injected 150 μL with Clodronate via tail vein, twice a week, for another 2 weeks accompanied by CCl_4_ stimulation. Finally, the liver and serum were collected for subsequent analysis.

To investigate the impact of blocking IL-11 *in vivo* (2023-12-GLK-39), the mice were treated with anti-IL11 nanobody F12 (n = 6) or the isotype antibody hFc (n = 6) at the dosage of 20 mg/kg via i.p. injection, three times a week. The two groups were received CCl_4_ at the same time as mentioned above. Apart from that, the littermates were administered with olive oil and saline as control (n = 7). After 4 weeks period, the mice were sacrificed, and the liver and serum were collected for subsequent analysis.

### Cells and culture conditions

All cells were cultured at 37 °C in humidified air with 5% (v/v) CO_2_. The LX-2 cells were purchased from the American Type Culture Collection (ATCC, USA), and were cultivated in RPMI-1640 medium containing 10% fetal bovine serum (FBS) and 1% penicillin/streptomycin (P/S). The immortalized mouse hepatic stellate cells (mHSCs) were purchased from Cellverse Co., Ltd (Shanghai, China). Cells were passaged regularly and grown to 80%-90% confluence before treatments.

### Statistical analysis

Statistical analysis was performed with GraphPad Prism 8 Software. Differences between two groups were determined using the two-tailed Student *t* test, and differences among three groups or more were evaluated by one-way analysis of variance. *P* < 0.05 was considered statistically significant.

## Results

### Overexpression of IL-11 in HSCs accelerates liver injury, fibrosis and inflammation in CCl_4_ mouse model

**Figure 1:**
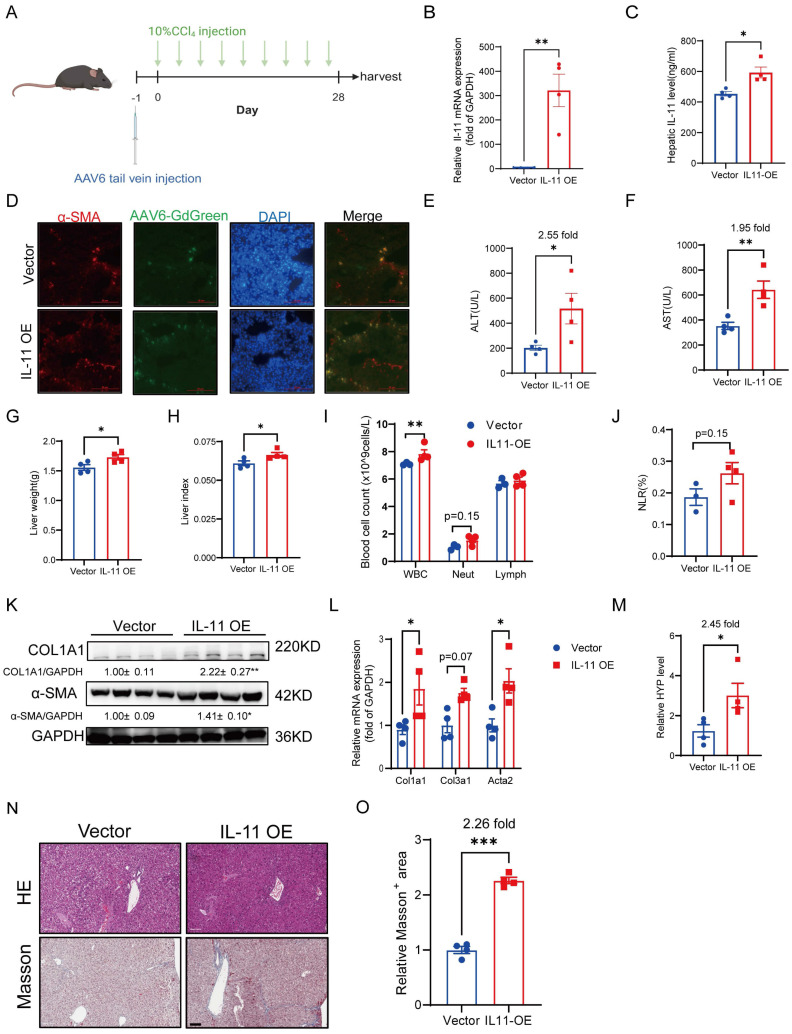
Overexpression of IL-11 in HSCs accelerates liver injury, fibrosis and inflammation. (A) Diagrams of HSC-specific overexpression of IL-11 and study design. (B-C) IL-11 overexpression efficiency in liver were confirmed at both mRNA level(B) and protein level(C) in liver lysates. (D) The α-SMA and DAPI staining of liver tissues. (E-F) ALT and AST levels in mouse serum. (G-H) Liver weight and liver index of mouse model. (I)The constitution of WBCs in peripheral blood. (J) NLRs presented by the number of neutrophils to lymphocytes ratio. (K) Protein level of COL1A1 and α-SMA in liver lysates. (L) mRNA levels of *Col1a1*, *Col3a1* and *Acta2* in liver tissues. (M) Content of hydroxyproline in mouse livers. (N) HE and Masson's trichrome staining of liver samples. Scale bar: 100 µm. (O) Quantification of Masson's Trichrome staining positive areas. Data are presented as the mean ± SEM. n = 4, *p < 0.05, ** *p* < 0.01, ***p < 0.001* vs.* Vector.

As reported, subcutaneous administration of rmIL-11 or specifically inducing IL-11 overexpression in hepatocytes by AAV8 in mice can cause liver damage with obvious profibrotic and proinflammatory phenotypes 18, 19. Since HSCs are the main cells expressing IL-11 in the liver 20, it is necessary to clarify whether IL-11 expression in HSCs is related to the progression of chronic liver fibrosis. In our work, AAV6 harboring *Il11* was injected into the tail vein to overexpress IL-11 in HSCs of the mice, accompanied by CCl_4_ stimulation either simultaneously or after 4 weeks of IL-11 overexpression (Fig. 1A, Fig. S1A). As shown in Figure 1B-D, AAV6 could transfer IL-11 in HSCs successfully. The increased levels of ALT and AST demonstrated that overexpressed IL-11 could exacerbate liver damage (Fig. 1E-F, Fig. S1B-C). We also found that IL-11 overexpression brought about a higher liver weight and hepatic index (Fig.1G-H). Results of hematological analysis showed that whole blood cells (WBC) counts in peripheral blood had a significant increase in IL-11 OE group accompanied by a slight promotion of neutrophils/lymphocytes ratio (NLR) (Fig. 1I-J), which indicated the augmentation of inflammation induced by IL-11 overexpression. Moreover, overexpressed IL-11 could remarkably increase the protein levels of COL1A1 and α-SMA as well as the mRNA levels of *Col1a1*, *Col3a1* and *Acta2* (Fig. 1K-L, Fig. S1D). Meanwhile, we also observed that IL-11 OE group had the higher hydroxyproline content (Fig. 1M, Fig. S1E). Finally, the representative HE and Masson's trichrome staining images showed in Figure 1N-O and Supplementary Figure 1F-G suggested that it could worsen the extent of liver fibrosis.

Briefly, these results confirmed that IL-11 in HSCs could accelerate liver injury, fibrosis and inflammation.

### IL-11 induces contractability and migration of HSCs *in vitro*

**Figure 2:**
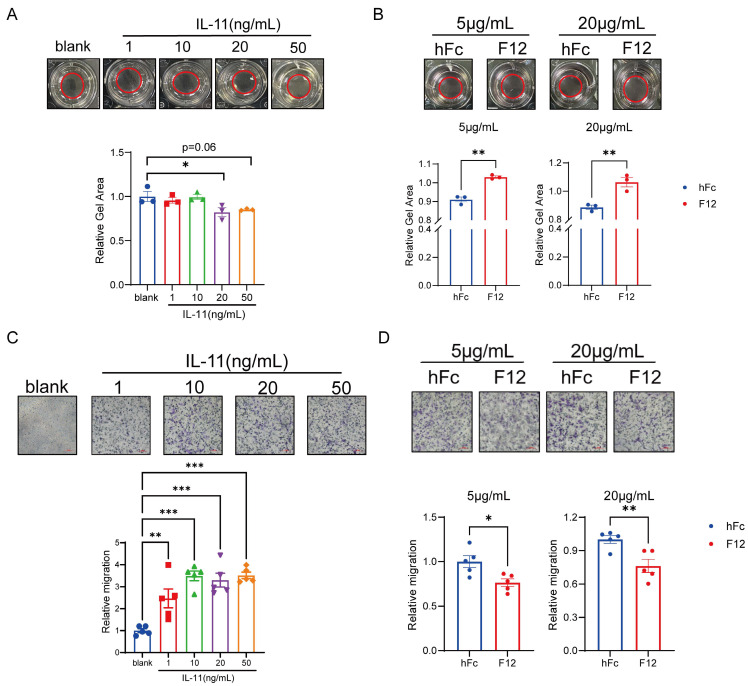
IL-11 induced contractability, migration and collagen synthesis of HSCs. (A) Dose-dependent collagen contraction of LX-2 induced by IL-11. (B) The contractability of LX-2 stimulated by IL-11(20ng/mL) with different concentration of hFc/F12. (C) Dose-dependent cell migration of LX-2 induced by IL-11. (D) Relative migration number of LX-2 stimulated by IL-11(10 ng/mL) with different concentration of hFc/F12. Data are presented as the mean ± SEM. N ≥ 3, *p < 0.05, **p < 0.01, ***p < 0.001* vs.* blank or hFc.

Considering the activated HSCs featured by the acquiring abilities to contract/migrate and the increase of collagen synthesis are the central drivers to lead to liver fibrosis 10, the role of IL-11 in HSCs during this process was our concern. It has been reported that IL-11 can induce the production of collagen I and α-SMA 21, we then examined the contractability and migration of HSCs under the stimulation of IL-11. F12 is an anti-IL11 nanobody generated by ourselves, which shared similar binding affinity to IL-11 between human and mouse (Fig. S2A-B). Next, we evaluated the positive effect of IL-11 and inhibitive effect of F12 on the phenotypic transition of LX-2 (human hepatic stellate cell line) from quiescent into activated state. IL-11 could shrink the size of collagen gel lattices in dose-dependent manner, which could be restored by F12 (Fig. 2A-B). Furthermore, IL-11 dramatically promoted LX-2 migration, reaching a plateau at 10 ng/mL, which were able to be reversed by F12 (Fig. 2C-D).

Collectively, these data demonstrated that IL-11 induced the contraction and migration abilities of HSCs, which could be limited by its antibody F12.

### IL-11 activates HSCs partly via GP130-SFK-YAP pathway

**Figure 3:**
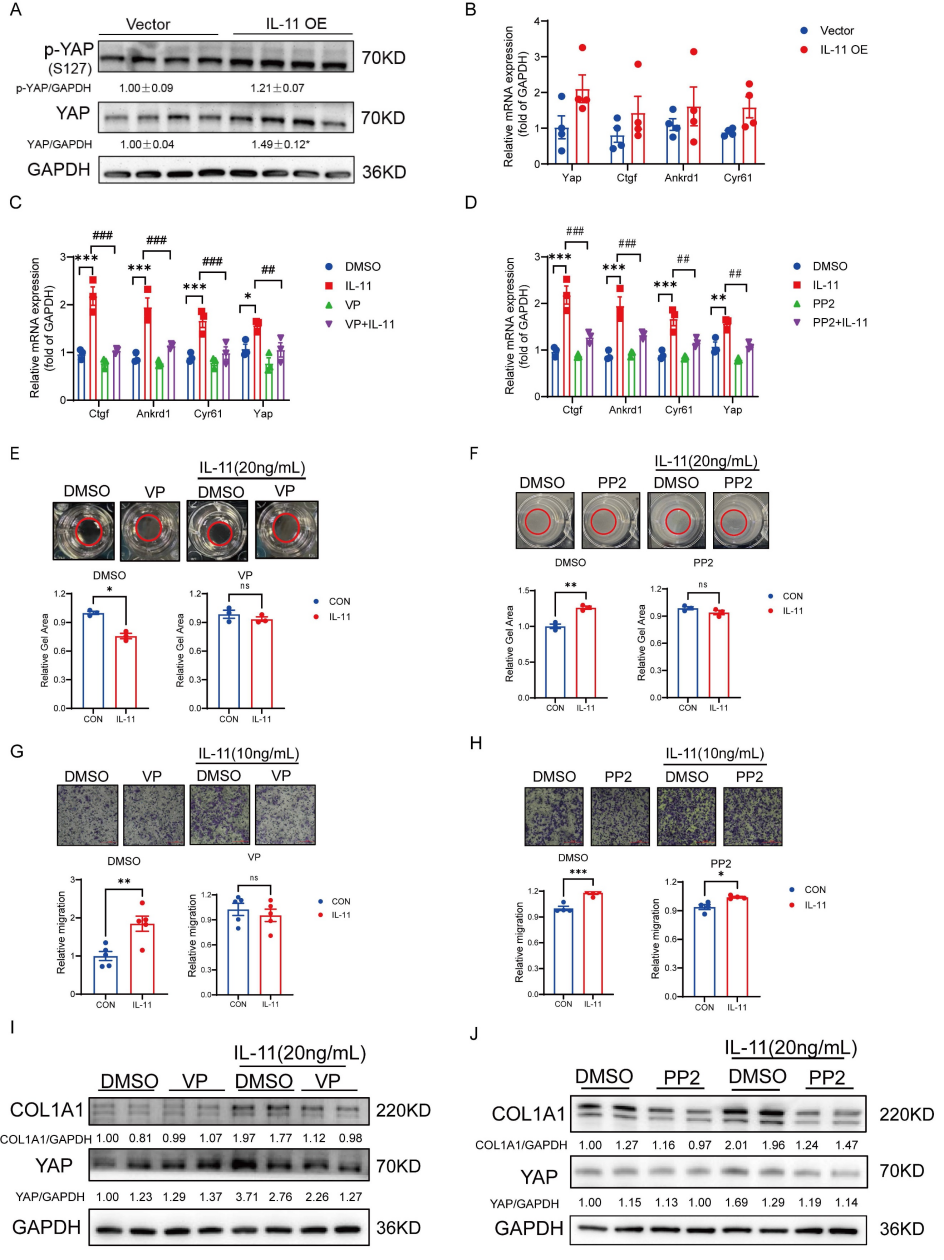
IL-11 activates HSCs partly via GP130-SFK-YAP pathway. (A) Protein level of YAP and p-YAP(S127) in liver lysates. (B) mRNA level of YAP and downstream genes (*Ctgf*, *Ankrd1*, *Cyr61*) in liver tissues. Effect of VP (100nM) and PP2 (5μM) on IL-11-induced the mRNA expression of YAP-target genes (C-D), gel contraction model (E-F), cell migration (G-H) and ECM accumulation (I-J) on LX-2. Data are presented as the mean ± SEM. n≥3, *p < 0.05, **p < 0.01, ***p < 0.001* vs.* Vector, DMSO or CON; #*p* < 0.05, ##*p* < 0.01, ###*p* < 0.001* vs.* IL-11.

Yes-associated protein (YAP) has been proved to control the quiescence-to-activation transition of HSCs by regulating the transcription of its downstream genes, including *Ctgf*, *Cyr61* and *Ankrd1*, and the aberrant activation of YAP signaling frequently leads to liver fibrosis 22, 23. Apart from the classical Hippo signaling pathway in regulating YAP, a GP130-SFK-YAP signaling axis had been reported to stimulate epithelial cell and hepatocytes proliferation for healing and maintain barrier function 24. Due to the fact that GP130 is the essential co-receptor for IL-11 signaling cascade, we wondered whether the activation of HSCs would be regulated via this pathway with IL-11 stimulation. At first, the mRNA and protein levels of YAP were tending to be raised in the IL-11 OE group compared with controls though the phosphorylation site S127 of YAP regulated by Hippo pathway was also induced, which suggested that IL11 could regulate YAP through multiple pathways, with the Hippo pathway being implicated but not the principal regulatory mechanism. (Fig. 3A). Meanwhile, the transcription levels of its target genes, *Ctgf*, *Cyr61* and *Ankrd1*, were increased as well (Fig. 3B). By co-treated with verteporfin (VP, YAP inhibitor) or PP2(SFK inhibitor), the target genes of YAP increased by IL-11 were depressed (Fig. 3C-D) in LX-2 cells, which hinted that IL-11 could regulated YAP signaling through GP130 and SFK indeed. The same phenomena were reproduced again in the gel contraction and migration assays of LX-2, where the abilities of contraction and migration stimulated by IL-11 were impeded in presence of VP, PP2 or Bazedoxifene (Baze, GP130 inhibitor) (Fig. 3E-H, Fig. S3A-B). Interestingly, we also discovered that VP, PP2 or Baze can inhibit the IL-11 induced the increase of COL1A1 protein (Fig. 3I-J, Fig. S3C), which suggested that YAP also played a key role in the impact of IL-11 on collagen synthesis besides the reported ERK pathway 18.

Above all, IL-11 may promote HSCs activation partly through GP130-SFK-YAP in terms of cell contractability, migration and collagen synthesis.

### Bulk sequencing analysis lightens the mechanism of how IL-11 facilitated the expression profile change of macrophages

**Figure 4:**
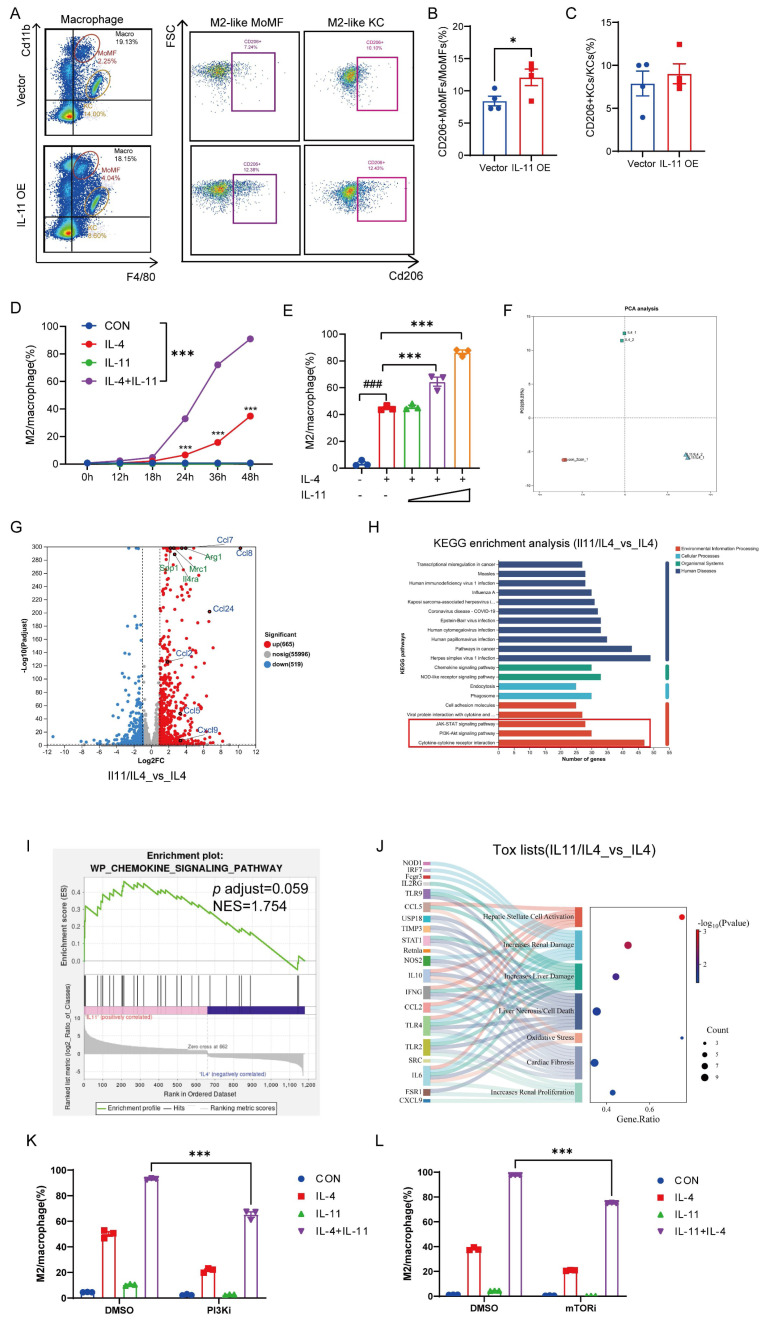
Bulk sequencing analysis lightens the mechanism of how IL-11 facilitated the expression profile change of macrophages. Representative histograms (A) and quantification analysis of CD206+MoMFs (B) and CD206+KCs (C) in IL-11 OE fibrotic mouse model (n = 4). The time- (D) and dose-(E) dependence of IL-11-faciliated BMDMs M2-like polarization by flow cytometry. IL-11(20 ng/mL) and/or IL-4(40 ng/mL) administrated BMDMs were collected for bulk sequencing. The PCA plot (F), differentially expressed genes by Volcano plot(G), KEGG analysis(H), GSEA analysis(I) and tox lists predicted by IPA(J) were presented. Flow cytometric analysis showing polarized BMDMs elicited by IL-11(20 ng/mL) and/or IL-4(40 ng/mL) could be suppressed by PI3Ki (LY294002,10 μM) (K) and mTORi (Rapamycin, 100 nM) (L) for 48 h. Data are presented as the mean ± SEM. N ≥ 3, *p < 0.05, **p < 0.01, ***p < 0.001* vs.* Vector or CON; #*p* < 0.05, ##*p* < 0.01, ###*p* < 0.001* vs.* blank.

In addition to the intracellular events, the extracellular signals from resident and inflammatory cells also modulate HSCs activation 10. Among them, the role of stimuli derived from macrophages in HSCs has made them as the key determinant of fibrosis resolution. Therefore, we detected the phenotypic change of macrophages when IL-11 overexpressed in HSCs in CCl_4_ mouse model. As shown in Figure 4A and Supplementary Figure 4A-C, the percentage of whole macrophages in the liver was virtually invariant between control (Vector) and IL-11 overexpressing (IL-11 OE) groups, whereas the percentage of Cd11b^hi^F4/80^int^ macrophages (MoMFs) had a rising trend and that of Cd11b^int^F4/80^hi^ macrophages (KCs) tended to sink in IL-11 OE group. Moreover, IL-11 overexpression promoted hepatic macrophages polarization to the M2-like phenotype (Cd11b^+^F4/80^+^CD206^+^), with a particularly significant increase in the proportion of M2 cells derived from in MoMFs (Fig. 4B-C). The same phenomenon was also replicated in *in vitro* experiments, in which BMDMs were treated with IL-4 to induce polarization to the M2-like phenotype, and IL-11 could amplify this effect in both time- and dose-dependent ways (Fig. 4D-E). Correspondingly, the mRNA levels of M2 markers, *Arg1* and *Mrc1*, were also further amplified by IL-11 under IL-4 stimulation (Fig. S4D). When IL-11 was saturated with its antibodies F12, these effects was restrained (Fig. S4E-G). Taken together, these data implied that the facilitative role of IL-11 in propelling the macrophages towards M2 phenotype both *in vitro* and *in vivo*.

To explore the mechanism of how IL-11 involved in prompting M2-like polarization of macrophages, we conducted a transcriptomic analysis of macrophages post-IL-11 and/or IL-4 treatment mentioned in Supplementary methods. Consequently, the PCA results depicted that IL-11 and IL-4 co-treatment changed the gene profile of macrophages induced by IL-4 alone, indicating that IL-11 treatment was capable of regulating M2-like macrophage polarization to a new state (Fig. 4F). There were 665 up-regulated and 519 down-regulated differential expression genes (DEGs) between IL-11/IL-4 group and IL-4 group. As shown in Fig. 4G, besides M2 marker genes (*Arg1*, *Mrc1*, *Spp1*, *Il4ra*), a series of chemokine genes (*Ccl2*, *Ccl5*, *Ccl7*, *Ccl8*, *Ccl24*, *Cxcl9*) were significantly elevated with addition of IL-11. KEGG enrichment analysis of up-regulated expression genes suggested that multiple signal pathways participated in the IL-11 plus IL-4 treatment, like cytokine-cytokine receptor interaction, PI3K-Akt signaling pathway, and JAK-STAT signaling pathway (Fig. 4H). Additionally, the results from GSEA revealed that the chemokine signaling pathway was significantly enriched after treatment with IL-11 (Fig. 4I). Moreover, IL-11 and IL-4 co-treatment of macrophages was predicted to cause HSCs activation and increase liver damage by IPA analysis (Fig. 4J). These results hinted us that PI3K-Akt and JAK-STAT signaling might participate in IL-11 induced M2-like polarization and chemokines production.

To verify the results of the bioinformatics analysis, PI3K inhibitor (PI3Ki, LY294002) or its downstream effector mTOR inhibitor (mTORi, Rapamycin) was added in the culture medium when induced M2-like polarization by IL-11 plus IL-4. As shown in Figures 4K-L and Supplementary Figures 4H-I, the percentage of M2 cells synergistically induced by IL-11 and IL-4 were obviously decreased, accompanied by the significant downregulation of M2 marker genes (*Arg1* and *Mrc1*).

In brief, IL-11induce M2-like macrophage differentiation synergistically with IL-4, which was dependent on PI3K-Akt-mTOR signaling pathway.

### IL-11 promotes TGF-β protein synthesis synergistically with IL-4 via PI3K-mTOR-S6K-S6RP

**Figure 5:**
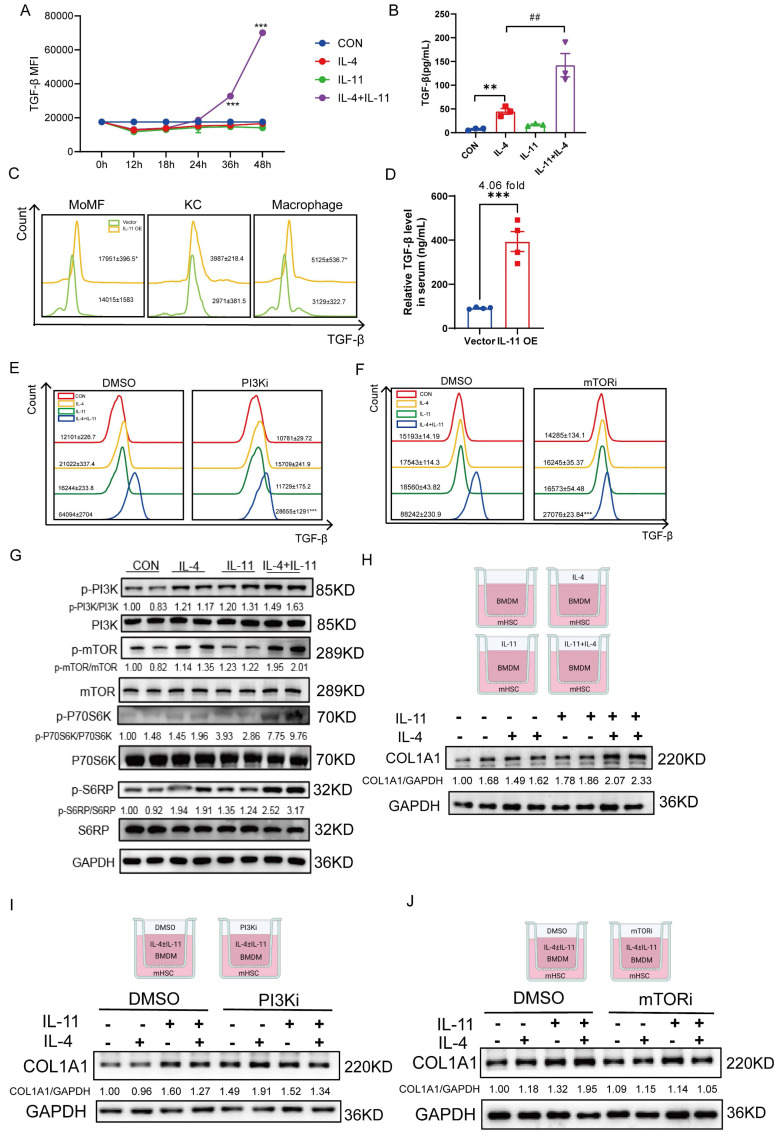
IL-11 promotes TGF-β protein synthesis synergistically with IL-4 via PI3K-mTOR-S6K-S6RP. (A) The time-dependence of IL-11(20ng/mL)-facilitated TGF-β protein expression in intracellular BMDMs. (B) TGF-β protein level in culture medium of BMDM detected by ELISA. (C) The TGF-β expression level of MoMF, KC and macrophages in liver of IL-11 OE mouse model. (D) TGF-β level in serum of IL-11 OE CCl_4_ model was quantified by ELISA. Flow cytometric analysis showing increased protein level of TGF-β elicited by IL-11(20ng/mL) and/or IL-4(40ng/mL) could be suppressed by PI3Ki (LY294002,10μM) (E) and mTORi (Rapamycin, 100nM) (F) for 48h. (G) Western blot analysis of PI3K-mTOR-P70S6K-S6RP signaling pathway protein expression in BMDMs with treatment of IL-11(20ng/mL) and/or IL-4(40ng/mL). (H)Western blotting of COL1A1 in mHSCs cocultured with IL-11(20ng/mL) and/or IL-4(40ng/mL)-treated BMDMs in Boyden chambers. Western blot analysis of COL1A1 in mHSCs cocultured with IL-11(20ng/mL) and/or IL-4(40ng/mL)-treated with PI3Ki (LY294002,10μM) (I) or mTORi (Rapamycin, 100nM) (J) BMDMs in Boyden chambers. Data are presented as the mean ± SEM. N ≥ 3, *p < 0.05, **p < 0.01, ***p < 0.001* vs.* Vector, CON or IL-11+IL-4 plus DMSO; #*p* < 0.05, ##*p* < 0.01, ###*p* < 0.001* vs.* IL-4.

Given macrophages, especially M2-like macrophages, are the major source of TGF-β which plays a crucial role in activating HSCs, we were curious of the effect of IL-11 on TGF-β expression. Firstly, the mRNA level of TGF-β remained irresponsive to IL-11 and/or IL-4 stimulation either in the sequencing data or the q-PCR result (Fig. S5A-B). Next, the levels of intracellular and extracellular TGF-β protein were determined by flow cytometry and ELISA, both of which could be obviously stimulated with cotreatment of IL-11 and IL-4 (Fig. 5A-B). Furthermore, the elevation of TGF-β protein level in serum (Fig. 5C) and hepatic macrophages, especially in MoMFs (Fig. 5D) of IL-11 OE group was observed, which was consistent with the *in vitro* results.

Since PI3K-Akt signaling plays an important role in M2 macrophages transition 25 and above results also showed that IL-11 could amplify M2-skewed macrophages under the stimulation of IL-4 by this pathway, we further investigated whether the effect of IL-11 on TGF-β depends on it. When PI3Ki or mTORi was introduced into the culture medium, the protein level of TGF-β induced by IL-11 and IL-4 was obviously decreased (Fig. 5E-F). p70S6K is a critical downstream effector of PI3K-Akt-mTOR pathway and its activation is tightly regulated by an ordered cascade of Ser/Thr phosphorylation events 26. Moreover, p70S6K is best known for its regulatory roles in protein synthesis and cell growth by phosphorylating its primary substrate, ribosomal protein S6(S6RP) 26. Therefore, we speculated that IL-11 regulated TGF-β synthesis through PI3K-mTOR-P70S6K-S6RP pathway, which were confirmed by the upregulation of phosphorylation of these factors following IL-11 treatment (Fig. 5G).

Then, we constructed a co-culture system which was consisted of BMDMs treated with IL-11 and/or IL-4 on the upper layer and mouse HSCs (mHSCs) on the lower layer. The western blotting results presented that secreted factors from polarized BMDMs could lead to activation of mHSCs, and this effect could be partially reduced probably because of the less secreted profibrotic factors, like TGF-β, from PI3Ki- or mTORi-treated BMDMs (Fig. 5H-J).

Taken together, we discovered that PI3K-mTOR-P70S6K-S6RP signaling played a crucial role in IL-11-induced TGF-β production in M2-skewed macrophages.

### CCL family is induced by IL-11 plus IL-4 mainly in STAT3-dependent manner

**Figure 6:**
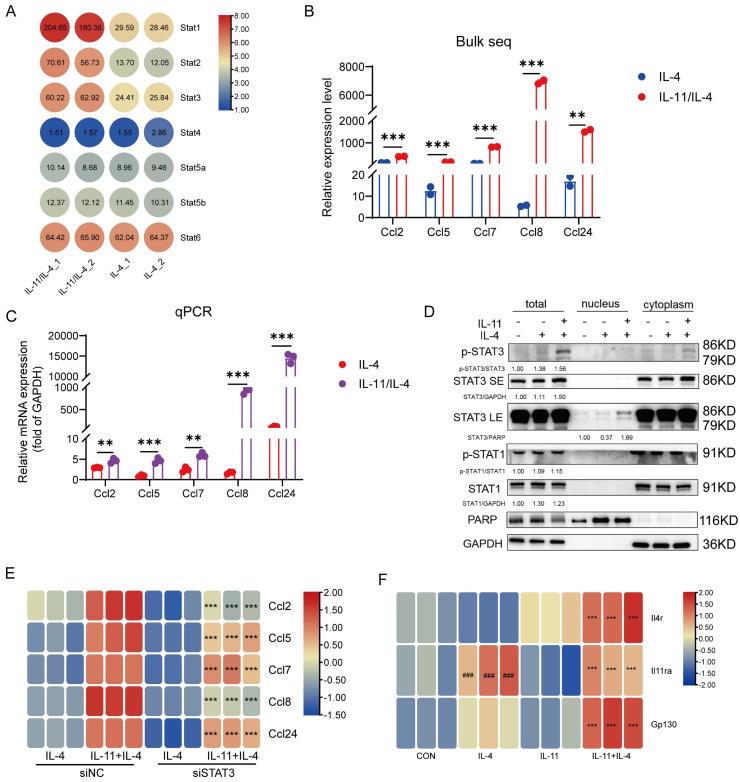
CCL family chemokines were induced by IL-11 plus IL-4 in STAT3-dependent manner. (A) The transcriptional level of STATs family in bulk sequencing result. (B) The transcriptional level of CCL family chemokines (Ccl2, Ccl5, Ccl7, Ccl8, Ccl24) in bulk sequencing result. (C) mRNA levels of Ccl2, Ccl5, Ccl7, Ccl8, Ccl24 were validated by q-PCR. (D) Western blot analysis of indicated proteins in the nucleus, cytoplasm or the whole cell lysates of BMDMs treated with IL-4 (40ng/mL) and/or IL-11 (20ng/mL) for 24 h. (E) Quantification of CCL family chemokines in BMDMs transfected with siNC (20nM) or siSTAT3 (20nM) for 48 h by q-PCR. (F) The transcriptional level of Il4r, Il11ra and Gp130 in BMDMs administrated with IL-11(20ng/mL) and/or IL-4(40ng/mL) for 48h. Data are presented as the mean ± SEM. N ≥ 3, *p < 0.05, **p < 0.01, ***p < 0.001* vs.* IL-4 or IL-11+IL-4 plus siNC; #*p* < 0.05, ##*p* < 0.01, ###*p* < 0.001* vs.* CON.

The aforementioned KEGG analysis were also enriched in JAK-STAT signaling cascade which is the main inflammatory signal transduction pathway 27. The relative level of STAT family members in our sequencing data were listed in Figure 6A, and STAT1/STAT2/STAT3 expression were elevated evidently. Meanwhile, the CCL chemokines, including Ccl2, Ccl5, Ccl7, Ccl8, Ccl24, were comparatively increased in IL-11 stimulation and this was also confirmed by q-PCR (Fig. 6B-C). We firstly checked if the elevated STATs were involved in the regulation of M2-like phenotype induced by IL-11 and IL-4. After inhibited STAT1 by its inhibitor (STAT1i), or knocked down STAT2 (Fig. S6A) or STAT3 (Fig. S6B) by corresponding siRNA in BMDM, there was no significant change in the percentage of M2 cells synergistically induced by IL-11 and IL-4, suggesting that STAT1, STAT2 and STAT3 hardly participated in the M2-like polarization (Fig. S6C-E).

Since it is seldom reported that STAT2 can regulate CCL family, we only detect the association of IL-11 modulating CCL family and STAT1/STAT3. By means of Western blotting, we noticed a promotion of p-STAT3 and STAT3 levels in both whole cell lysates and nucleus in response to IL-11 stimulation, while no alternation was observed in regard to the level of STAT1 and p-STAT1 (Fig. 6D). The results of knocking down STAT3 with siRNA inhibiting the response to IL-11 significantly was further verified that STAT3 participated in regulating the IL-11-stimulated CCL family factors (Fig. 6E). In addition, as the PI3K-Akt signaling pathway could shape the macrophage state, we wondered if it also participated in CCL factors production of IL-11 and/or IL-4 treated macrophages. As shown in Supplementary Figures 6F, the CCL factors only could be suppressed partially by PI3Ki incubation under treatment of IL-11 and/or IL-4, indicated that STAT3 pathway was the main pathway responsible for IL-11 regulation of the CCL family. Since CCL factors could stimulate HSCs directly as well, the results of co-culture system showed that the CCL chemokines production mediated by STAT3 also participated in regulating the HSCs activation (Fig. S6G).

### Synergistic effect of IL-4 and IL-11 might be related to the expression of their receptors

As mentioned above, we discovered that IL-11 could amplify the phenotype transformation of macrophages to M2 promoted by IL-4, including an increase in M2 classic marker genes, CCL family and TGF-β. We wondered why IL-11 alone cannot exert these effects. Considering that IL-4 and IL-11 depend on their own receptors or co-receptors to transmit signaling, we determined the expression of IL-4 receptor (Il4r), IL-11 receptor subunit alpha (Il11ra), and IL-6 cytokine family signal transducer (Il6st, also named Gp130) on macrophages under the circumstance of IL-4, or IL-11, or IL-4/IL-11. The q-PCR results showed that IL-11 could enhance mRNA level of Il4r, while IL-4 could promote Il11ra mRNA level at the same time, and co-stimulation of IL-4 and IL-11 eventually made the mRNA expression of Gp130 grew up (Fig. 6F). These results posited that IL-11 and IL-4 signaling had the mutual interference by upregulation the receptor of counterpart. To conclude, we supposed that the positive regulation of Il11ra and Gp130 by IL-4 might account for the synergistic effect of IL-4 and IL-11 on M2-like polarization and related factors production.

### Macrophage depletion alleviates HSCs-derived IL-11-mediated hepatic fibrosis

**Figure 7:**
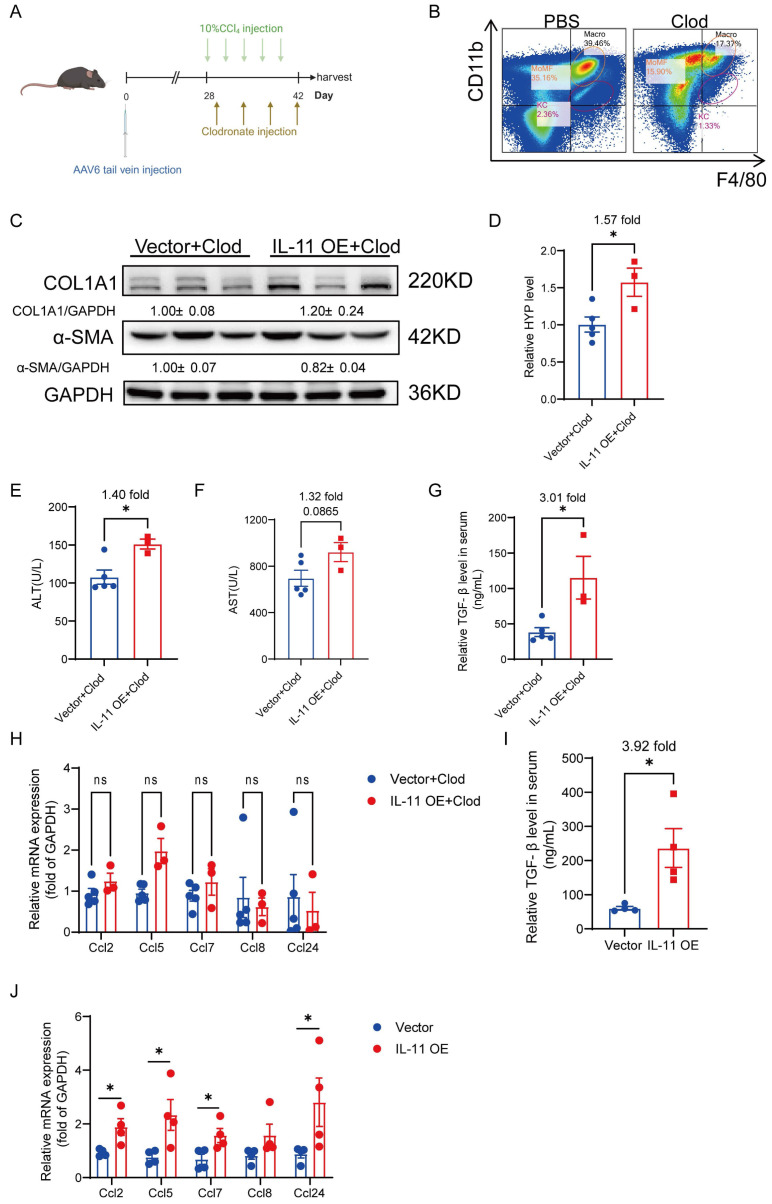
Macrophage depletion could alleviate HSC-derived IL-11-mediated hepatic fibrosis. (A) Scheme of macrophage depletion in IL-11 OE and Control group. (B) Macrophage-depleted efficiency was analyzed by flow cytometry for F4/80 and Cd11b. (C) Western blot analysis of COL1A1 and α-SMA in mouse livers. (D) Content of hydroxyproline in mouse livers. ALT(E) and AST(F) level in peripheral serum. (G) TGF-β level in serum of IL-11 OE CCl_4_ model treated with Clod was detected by ELISA. (H) mRNA levels of* Ccl2*, *Ccl5*, *Ccl7*, *Ccl8* and *Ccl24* in liver tissues of IL-11 OE CCl_4_ model treated with Clod. (I) TGF-β level in serum of IL-11 OE CCl_4_ model was detected by ELISA. (J) mRNA levels of* Ccl2*, *Ccl5*, *Ccl7*, *Ccl8* and *Ccl24* in liver tissues of IL-11 OE CCl_4_ model. Data are presented as the mean ± SEM. n≥3, *p<0.05, **p<0.01, ***p<0.001* vs.* Vector+Clod or Vector.

To figure out the contribution of the crosstalk between HSCs and hepatic macrophages in IL-11 induced fibrosis *in vivo*, we depleted macrophages by injecting clodronate liposome (Clod) during CCl_4_-induced fibrogenesis after IL-11 overexpression in HSCs (Fig. 7A). There were approximately 56% of macrophages deleted, which was determined by flow cytometry (Fig. 7B). Compared with the mice in the same batch that did not clear macrophages (Fig. S1), the reduction of macrophages in the liver could weaken the degree of liver fibrosis induced by IL-11 overexpression, manifested as an attenuation of increased COL1A1 and α-SMA (Fig. 7C), and a decrease in HYP elevated by IL-11 from 2.43-fold without clearance to 1.57-fold after clearance (Fig. 7D). What's more, the ALT and AST levels in serum were also not increased strikingly as before (Fig. 7E-F). We discovered that IL-11 could facilitate to increase M2 macrophage differentiation and promote TGF-β and CCL family, which were further verified in this macrophage depletion experiment. Both serum TGF-β and hepatic CCLs rose up in IL-11 overexpression group could be alleviated by macrophage depletion *in vivo* (Fig. 7G-J), which suggested macrophage as a potent paracrine target of IL-11-faciliated liver fibrosis, except for the autocrine effect of IL-11 on HSCs.

### Blockade of IL-11 signaling with nanobody alleviates CCl_4_-induced liver injury

**Figure 8:**
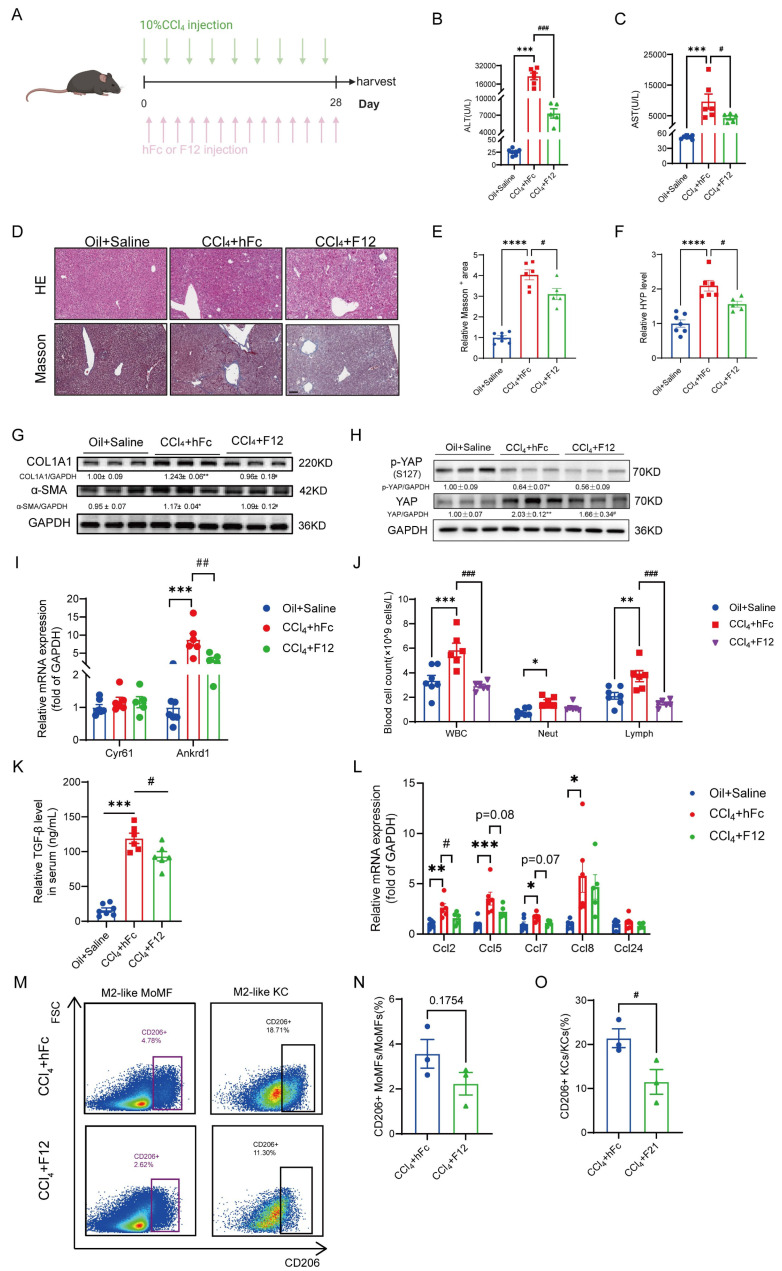
Blockade IL-11 signaling with nanobody could alleviate CCl_4_-induced liver injury. (A) Schematic view of the experimental design. ALT(B) and AST(C) level in mouse serum. (D) Paraffin sections were stained with HE and Masson's staining. Scale bars: 100μm. Hepatic fibrosis was determined by quantification of Masson+ area(E) and content of hydroxyproline(F). (G) Western blot analysis of COL1A1 and α-SMA in mouse livers. Relative protein level of YAP and p-YAP(S127) (H) and the transcriptional expression of its target genes: Ankrd1 and Cyr61(I). (J) Hematological analysis results of murine peripheral blood. (K) Serum TGF-β level detected by ELISA. (L) mRNA level of CCL factors in murine liver. Representative flow cytometric images (M) and quantification of CD206+MoMFs (N) and CD206+KCs (O) in mouse livers. Data are presented as the mean ± SEM. n ≥ 3, *p < 0.05, **p < 0.01, ***p < 0.001* vs.* Oil+Saline; #*p* < 0.05, ##*p* < 0.01, ###*p* < 0.001* vs.* CCl_4_+hFc.

As reported, blockade of IL-11 signaling in diet-induced MAFLD mouse model can relieve liver fibrosis 17, 18, we evaluated the benefit of IL-11 nanobody on the CCl_4_-induced liver injury to further highlight the potent role of IL-11 in fibrogenesis (Fig. 8A). Firstly, the serum ALT and AST post 2-week intervention of F12 lowered obviously compared with the isotype-treated group (Fig. 8B-C). Furthermore, HE and Masson staining showed that F12 administration effectively narrowed fibrotic area (Fig. 8D-E). Similarly, we also observed that F12 significantly suppressed the hydroxyproline content and reduced the protein level of fibrotic markers (COL1A1 and α-SMA) (Fig. 8F-G). Apart from that, the expression of YAP and its target genes were declined in the liver though the level of p-YAP(S127) were rarely changed, suggesting that F12 inhibiting YAP pathway probably contributed to the suppression of HSCs (Fig. 8H-I). Concurrently, the hematological results indicated that the number of white blood cells, neutrophils and lymphocytes was downregulated in F12-administrated group (Fig. 8J). In addition, the elevated TGF-β level in serum caused by CCl_4_ could be limited by administration of F12 as well as CCL chemokines in the liver (Fig. 8K-L). Finally, the changes of macrophages in liver were detected by flow cytometry, the results of which presented that anti-IL-11 lowered, or trended toward lowering M2-like MoMFs, and M2-like KCs especially (Fig. 8M-O). All of the above results were consistent with our findings in the IL-11 overexpression model, suggesting that F12 is a promising candidate to brake inflammation and fibrosis in the liver fibrogenesis.

## Discussion

In the past years, the epidemiological researches revealed that liver fibrosis mainly results from three types chronic liver injury: hepatic virus infection, chronic chemical liver injury especially caused by alcohol abuse, and metabolic dysfunction-associated steatotic liver disease (MASLD) 28. Nevertheless, the development of efficient antifibrotic therapies has still been challenging. Although there are numerous promising results from preclinical studies, only few are translated to treat human disease in clinical trials. Consequently, exploration on the mechanism of key regulatory elements prompting hepatic fibrosis will facilitate the development of novel therapeutic strategies. It is widely recognized that the transition of HSCs-to-myofibroblasts is the pivotal process during liver fibrosis, therefore, discovery of the key events involved and elucidation of underlying mechanism is crucial. In our work, HSCs intrinsic IL-11 is proved to be a hazardous determinant in the pathological progression of hepatic fibrosis. On the one hand, the direct activation on HSCs is relied on GP130-SFK-YAP pathway; on the other hand, upregulated M2-like macrophages polarization in the liver promotes profibrotic inflammatory microenvironment. Application of an anti-IL-11 nanobody both elicited considerable anti-fibrosis effects and suppressed the polarization of hepatic macrophages.

IL-11 is identified from bone marrow stromal cells at first 29 and developed to treat patients with thrombocytopenia resulted from exposure to chemotherapy. Recently, IL-11 has been discovered as a critical downstream factor of TGF-β which will lead to multiple organ fibrosis, including liver. The activation mechanism of IL-11 on fibroblasts among multi-organ has been reported to be associated with ERK signaling pathway 16, though other signaling pathways can be activated by IL-11 including JAK-STAT3 pathway, PI3K-Akt-mTOR pathway and Notch signaling as well as YAP 24, 30, 31. YAP is a core transcriptional factor in modulating metabolic cycles, cell proliferation, inflammatory factor expression and fibroblasts activation 32. According to the clinical TCGA dataset, the expression level of IL-11 and YAP-related genes were positively correlated in Liver hepatocellular carcinoma (LIHC) patients (data not shown). In our work, we identified YAP pathway is involved in IL-11-medidated transition of HSCs-to-myofibroblasts, which might not only be related to liver fibrosis, but also to the cancer-associated fibroblasts in hepatocellular carcinoma.

The biological functions of IL-11 in the liver are currently mainly focused on the stromal cells, like hepatocytes and HSCs, due to that IL-11RA is predominantly distributed on stromal cells compared with immune cells 18. However, the expression of IL-11RA also vary depending on whether healthy or diseased cells were studied 33, it is thus necessary to investigate the effect of IL-11 on the NPCs in the liver during fibrogenesis for the important role of the surrounded inflammatory environment in the progress. Macrophages as the largest innate immune cells play the determinant role in HSC activation 11, which attracted our attention. We have found for the first time IL-11 could promote M2 polarization in CCl_4_ mouse model and synergistically increase the percentage of M2 polarization in BMDM with IL-4. It is common for IL-6 family factors to polarize macrophages to a M2-like phenotype. IL-6 has been reported to enhance primary human monocyte-derived macrophages (hMDMs) M2 polarization alone or in combination with IL-4 34. Another IL-6 family member OSM could directly modulate hepatic macrophages towards a profibrotic state 35. Our results also indicate the function of IL-11 as a member of the IL-6 family in regulating macrophage polarization. However, its effect on polarization cannot be exerted alone and requires synergistic action with other stimuli such as IL-4.

As reported, TGF-β protein level is repressed in diet-induced live fibrotic mouse model when administrated with IL-11/IL-11RA neutralizing antibody 18, which is also confirmed in our results once again. Moreover, we expound that IL-11 promotes synthesis of TGF-β protein through PI3K-mTOR signaling pathway in macrophages. The reduction of TGF-β protein involves in its transcription and post transcriptional regulation. It has been reported that TGF-β transcription is regulated by MAPKs including p38, ERK, and JNK, whereas TGF-β translation required activation of Rho GTPase, PI3K-Akt-mTOR-eIF4E 36. In our work, we observed that increased TGF-β protein was irrelevant to TGF-β mRNA level when exposing to IL-11 treatment and this situation could be reversed by inhibition of PI3K and mTOR. Furthermore, we speculate that PI3K-mTOR-S6K-S6RP might participate in regulation TGF-β translation. The state of phosphorylated S6RP was highly enriched at the initiation of translation, but it would go through progressive dephosphorylation with the peptide elongation. Researches about translation selectivity of S6RP phosphorylation indicated that mRNAs with shorter Open Reading Frames (ORFs) tended to be affected by S6RP phosphorylation in a deeper way 37, which may provide a possible explanation for the regulation of IL-11 on TGF-β translation. Despite that, how Il-11 regulates PI3K and whether eIF4E involves in this regulation are still unknown, which needs further investigation.

According to ontogenetic origin, liver macrophages mainly comprise tissue-resident Kupffer cells (KCs) and monocyte-derived macrophages (MoMFs). In our *in vivo* results, IL-11 overexpression can increase the percentage of CD206^+^ cells in all macrophages including KCs and MoMFs, whereas F12 treatment can inhibit it. By utilizing chlodronate to deplete the whole macrophages in the liver, IL-11 OE-induced pro-fibrogenesis was partially eliminated. As reported, KCs and MoMFs exhibit heterogeneity in several ways including phenotypes and functions, and they could mutually transform in mouse models 38, 39. Our data do not clarify the contribution of KCs and MoMFs to the regulation of liver fibrosis separately and further study such as selective depletion of KCs or specific chemokine inhibitors targeting MoMFs may be necessary to understand their roles in the process of liver fibrosis.

Inevitably, our present study has some limitations. Firstly, the liver immune environment is highly complicated and cell-cell interaction builds up a sophisticated network. HSCs is a hub of intrahepatic signaling by HSC-derived stellakines, among which IL-11 is a key member. Therefore, it might participate in the crosstalk between HSCs and other NPCs except macrophages 20. Moreover, PI3K-mTOR signaling is a multifaceted pathway which could regulate cell cycle, energy conversion and cell survival. IL-11-driving macrophage polarization is tightly correlated with this pathway. Since PI3K-mTOR-mediated metabolic reprogramming also engages in macrophage polarization 40, whether the change of metabolic state involved in IL-11-skewed polarization needs further study. Lastly, the synergy effect of IL-4 and IL-11 on transition of macrophage M2-like phenotype relied on collaborative growth of receptors, but the regulation of receptors expression needs additional research.

## Conclusions

Overall, our work elucidates that IL-11 is a critical factor to induce liver fibrosis by activating HSCs through GP130-SFK-YAP pathway, and promoting fibrogenesis by inducing TGF-β and chemokines expression through the M2-like phenotypic change of macrophage.

## Supplementary Material

Supplementary methods, figures and tables.

## Abbreviations

HSCs: Hepatic stellate cells
BMDMs: Bone-derived macrophages
ECM: Extracellular matrix
NPCs: Non-parenchymal cells
NLR: neutrophil-to-lymphocyte ratio
MoMFs: Monocyte-derived macrophages
KCs: Kupffer cells
Clod: Clodronate
ALT: Alanine aminotransferase
AST: Alanine aminotransferase
VP: Verteporfin
