# Supplementary material for: Hepatic Stellate Cell-derived IL-11 Exacerbates Liver Fibrosis via Interplay between HSCs and Macrophages
Source: Int J Biol Sci. 2026 Jan 1;22(1):126–41. doi: 10.7150/ijbs.119659 (PMC12681746; doi:10.7150/ijbs.119659)
Supplement: Supplementary file 1 — Supplementary methods, figures and tables. [file ijbsv22p0126s1.pdf]

Hepatic stellate cells-derived IL-11 exacerbates liver fibrosis via interplaying between HSCs and macrophages

Yu Zhang[1,2], Fangfang He[1,2], Mozi Lei[1,2], Wenhui Fan[3,1], Xingyu Liu[3,1], Ying Tao[3,1], Weinan Wang[3,1], Bingshun Wang[4,*], Likun Gong[1,2,3,5*], Jing Chen[1,2,3*]

1. State Key Laboratory of Drug Research, Shanghai Institute of Materia Medica, Chinese Academy of Sciences, Shanghai, China.
2. School of Pharmacy, University of Chinese Academy of Sciences, Beijing, China.
3. School of Chinese Materia Medica, Nanjing University of Chinese Medicine, Nanjing, China.
4. Department of Biostatistics, Clinical Research Institute, Shanghai Jiao Tong University School of Medicine, Shanghai, China.
5. China-Serbia "Belt and Road" Joint Laboratory for Natural Products and Drug Discovery, Shanghai Institute of Materia Medica, Chinese Academy of Sciences, Shanghai, China.

Table of contents

Supplementary materials and methods 2
Figure S1 6
Figure S2 7
Figure S3 8
Figure S4 9
Figure S5 11
Figure S6 12
Table S1 13
Table S2 14
Table S3 14
References 15

Supplementary Materials and methods

Hematoxylin-Eosin staining and Masson's trichrome staining

Conventional hematoxylin-eosin (H&E) staining and Masson staining was performed according to established protocols(1). Five fields from each slide were selected randomly, and positive area was measured quantitatively using ImageJ software.

Immunofluorescence staining

The expression and localization of AAV2/6 were investigated by immunofluorescence. Meanwhile, Rabbit anti-mouse α-SMA antibody (A7248, 1:200; Abclonal) was used to stain activated hepatic stellate cells in liver sections. The live slides were incubated with the following secondary antibodies for 1 h: FITC-labeled goat anti-rabbit IgG (H+L (1:500)). Nuclei were stained with DAPI for 3 min. Finally, the cells were observed under a fluorescence microscope.

Biochemical and hematological analysis

Serum alanine aminotransferase (ALT), and aspartate aminotransferase (AST) were determined by a fully automated biochemical analyzer from Roche (Cobas C501).

Hepatic hydroxyproline (HYP) content test

Hydroxyproline (HYP) is one of the main components of collagen and serves as a marker of collagen accumulation in the liver(2). The content of HYP in livers of model mice were measured by Hydroxyproline Detection Kit (Solarbio, Beijing,China).

Bone marrow-derived macrophages (BMDMs) isolation

Recombinant M-CSF (PeproTech, USA) were used to differentiate bone marrow derived macrophages (BMDMs) as previously described(3). Briefly, femurs and tibias were carefully dislodged from sacrificed 8~12 weeks-old male C57 mice in a laminar flow hood. Bone marrow cells were then flushed out using 30G needle on a 20 mL syringe filled with DMEM. After red blood lysis using red blood cell lysis buffer (Yeasen, 40401ES60), cells were washed with PBS and seeded in DMEM medium containing 20 ng/mL M-CSF and

10% fetal bovine serum (FBS). After 4-day cultivation, cells were regarded as fully differentiated BMDMs.

Collagen gel contraction assay

This experiment was carried out as previously described(4). The collagen gel was prepared in pre-cooled 24-well plates at 4°C. NaOH (0.1 mol/L) was used to adjust the pH and 10× PBS was used to adjust the solution to physiological strength. The mixed solution (300 μL) was added to each well and incubated at 37 °C for 1 h to allow gelatinization. And LX-2 cells were seeded on the gel overnight for adhesion. The next day, different treatments were added into the culture medium and the gels were dissociated with a pipette tip. The floating gels were cultured for up to 2 days, and the ability of LX-2 to contact the gels was quantified ImageJ software.

Migration assays

0.2 ml of LX-2 (6×10^4 cells) were placed on the upper layer of Transwell inserts (8 μm, #3422, Corning, Kennebunk, ME, United States) containing different treatment of rhIL-11 and hFc/F12. After incubation in a 37 °C incubator with 5 % CO_2 for 24 h, a cotton swab was used to wipe off the cells in the insert. The insert was fixed with 4 % polyoxymethylene for 30 min and then stained with 1% crystal violet (MeilunBio, MA0148) for 10 min. Images were captured under an inverted microscope in more than five random fields, and then the cell number was calculated(5).

Co-culture assays

0.6 ml of mHSC were layered on a 24-well plate (1.6×10^5 cells/ml), and the Transwell inserts (0.4 μm, #3413, Corning, Kennebunk, ME, United States) were placed into a 24-well plate containing 0.2 ml of BMDMs(5×10^4 cells) received different treatment. After incubation in a 37 °C incubator with 5 % CO_2 for 24 h, mHSCs on the lower layer were collected for western blotting analysis.

RNA interference

BMDMs were cultured in 12-well plate, then the cells were transfected by Lipofectamine RNAiMAX (Invitrogen, USA) containing siControl or siSTAT3/siSTAT2 for 48 h. The siRNA sequence was listed in Table S2.

Western blotting

The protein in whole-cell lysates or liver homogenates were separated by electrophoresis. After being electro-transferred to PVDF membranes (Millipore, USA), the proteins were detected using the antibodies. The antibodies used in this research are listed in Table S3. The signals were detected using the Electrochemiluminescence (ECL) substrate (ShareBio, SB-WB012) and images were obtained by Automatic Chemical Imaging System (Tanon 5200, Shanghai, China).

q-PCR

Total RNA was extracted from cells or liver homogenates using Trizol reagent (Takara, Japan) according to the manufacturer's protocol, which then was reverse-transcribed using the PrimeScript RTMaster Mix (Takara), followed by qPCR with Hieff® qPCR SYBR Green Master Mix (Yeasen, China) and detected by 7500 FastReal-Time PCR System (Thermo Fisher Scientific, USA). The relative mRNA level was calculated by the 2(−ΔΔCt) method with GAPDH as an internal control. Primers used in this study were listed in Table S1.

ELISAs

Liver IL-11 concentrations were determined by a mouse IL-11 ELISA kit (#JL19247, Jianglaibio) according to the manufacturer's instructions. An ELISA kit (#EK981, Multi sceinces bio) was used to detect TGF-β level in the culture medium of BMDMs. The culture medium was collected and centrifuged at 3000g to remove cell debris, then tested based on the manufacturer's instructions.

Flow cytometry analysis

A liver dissociation kit (Miltenyi,130-105-807) and gentle MACS (Miltenyi) were used to digest the tissues into single-cell suspensions according to the manufacturer’s instructions.

Then, we followed the instructions of the Debris Removal Solution kit (Miltenyi, 130–109–398) to remove cell debris. The pellet was resuspended in 4 ml of red blood cell lysis buffer (Yeasen, 40401ES60) to lyse red blood cells. the cells were blocked with 4% FBS and anti-CD16/CD32 (553141, BD Biosciences, Franklin Lake, NJ, USA), incubated with surface marker antibodies for 20 min at 4 °C and then permeabilized with BD Cytofix/Cytoperm buffer (554714) before intracellular labeling antibodies were added for 30 min at 4 °C. Flow cytometry analysis was performed using ACEA NovoCyte and data processing was done through NovoExpress software (version 1.6.1). The gating strategy are shown in Fig.S5. Antibody staining was performed following the manufacturer's recommendations. Antibodies used were listed in Table S3.

Bulk-seq analysis

RNA isolation, transcriptome libraries construction, sequencing and basic data analysis were conducted by MajorBio. Based on the RNA-seq raw data, differential expression was evaluated with DESeq. A fold-change of 2:1 or greater and a false discovery rate (FDR)-corrected P-value of 0.05 or less were set as the threshold for differential genes. Chemokine signaling scores are defined as the mean log2(fold-change) among all genes in each gene signature list from Gene Set Enrichment Analysis (GSEA) datasets. Tox lists were predicted by IPA software.

Statistical analysis

Statistical analysis was performed with GraphPad Prism 7.0 (GraphPad Software Inc, La Jolla, CA, USA). All data are presented as mean ± SEM. Differences between two groups were determined using the two-tailed Student *t* test, and differences among three groups or more were evaluated by one-way analysis of variance, followed by Tukey's *post hoc* test for data meeting homogeneity of variance or with Tamhane's T2 analysis for data of heteroscedasticity. $P < 0.05$ was considered statistically significant.

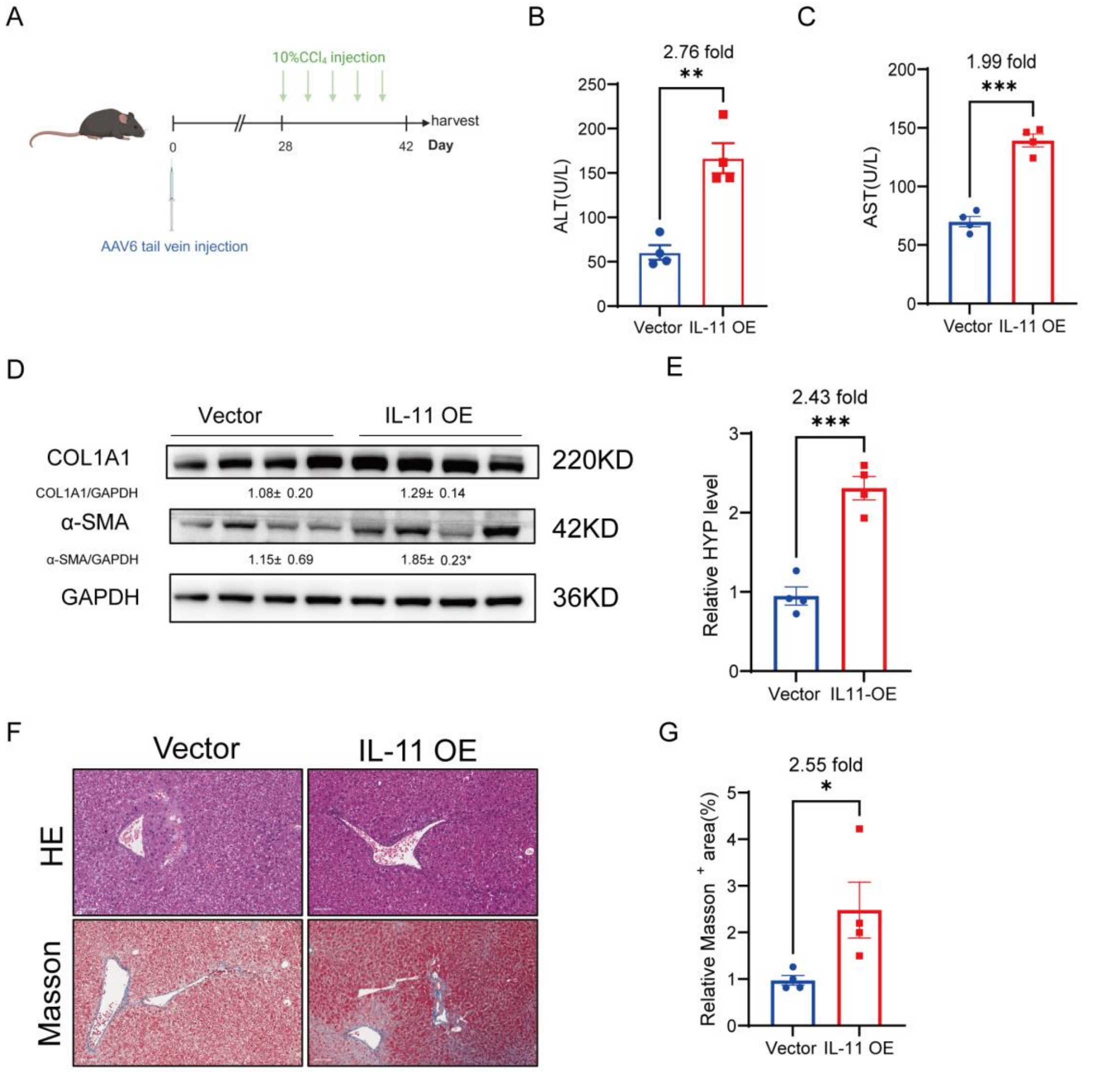

Supplementary Figure 1. Liver injury, fibrosis and inflammation could be aggravated in IL-11 pre-overexpressed mouse model. (A) Scheme of pre-overexpressed IL-11 in CCl_4 murine model. (B-C) ALT and AST levels in mouse serum. (D) Protein level of COL1A1 and α-SMA in liver lysates. (E) Content of hydroxyproline in mouse livers. (F) Representative HE and Masson's trichrome staining of liver samples. Scale bar: 100 μm. (G) Masson+ area per field was quantified. Data are presented as the mean ± SEM. n=4, **p*<0.05, ** *p*<0.01, ****p*<0.001 *vs.* Vector.

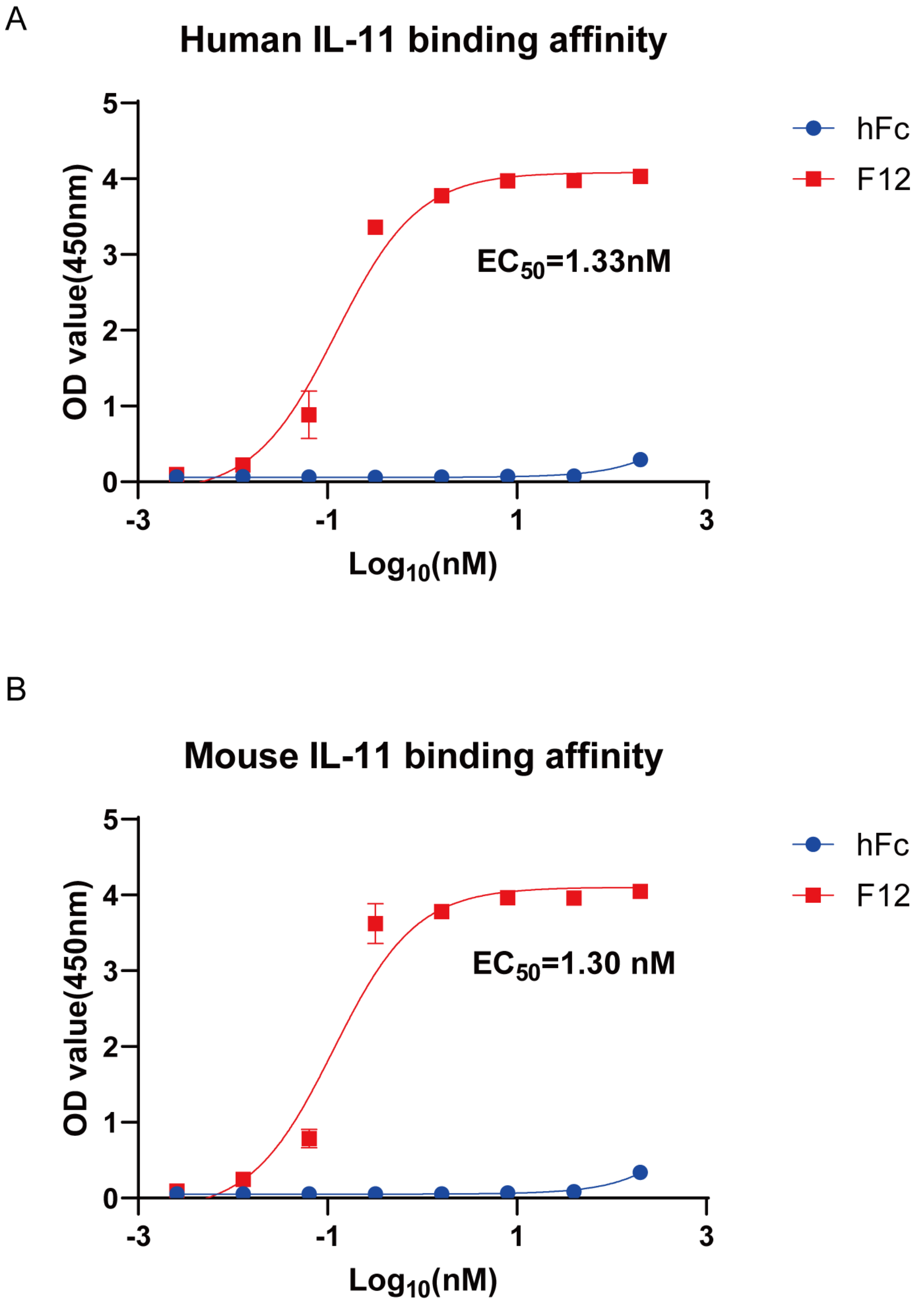

Supplementary Figure 2. The binding affinity of hFc and F12 towards human IL-11 and mouse IL-11. (A) Comparison of hFc with F12 in human IL-11 binding affinity detected by ELISA. (B) Comparison of hFc with F12 in mouse IL-11 binding affinity detected by ELISA.

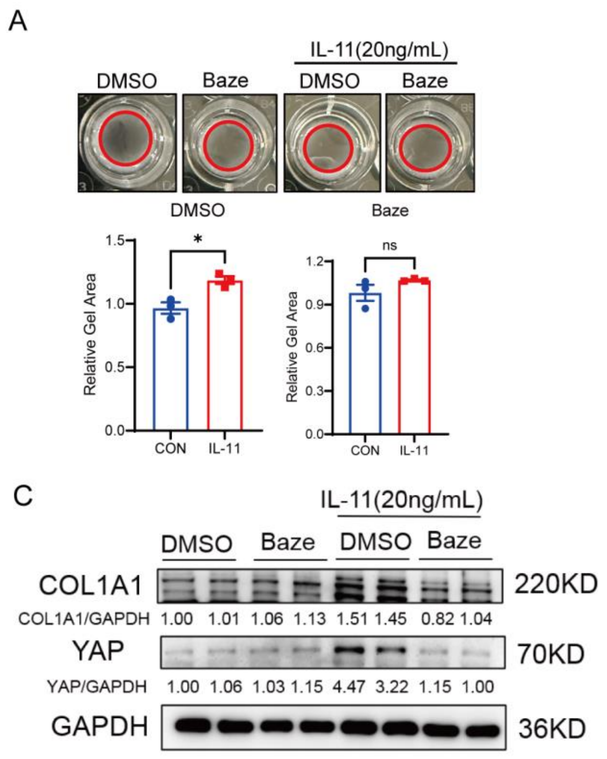

Supplementary Figure 3. IL-11 activates HSCs via YAP dependent on GP130. Effect of Bazedoxifene(10μM) on IL-11-induced gel contraction model (A), cell migration (B) and ECM accumulation (C) on LX-2.

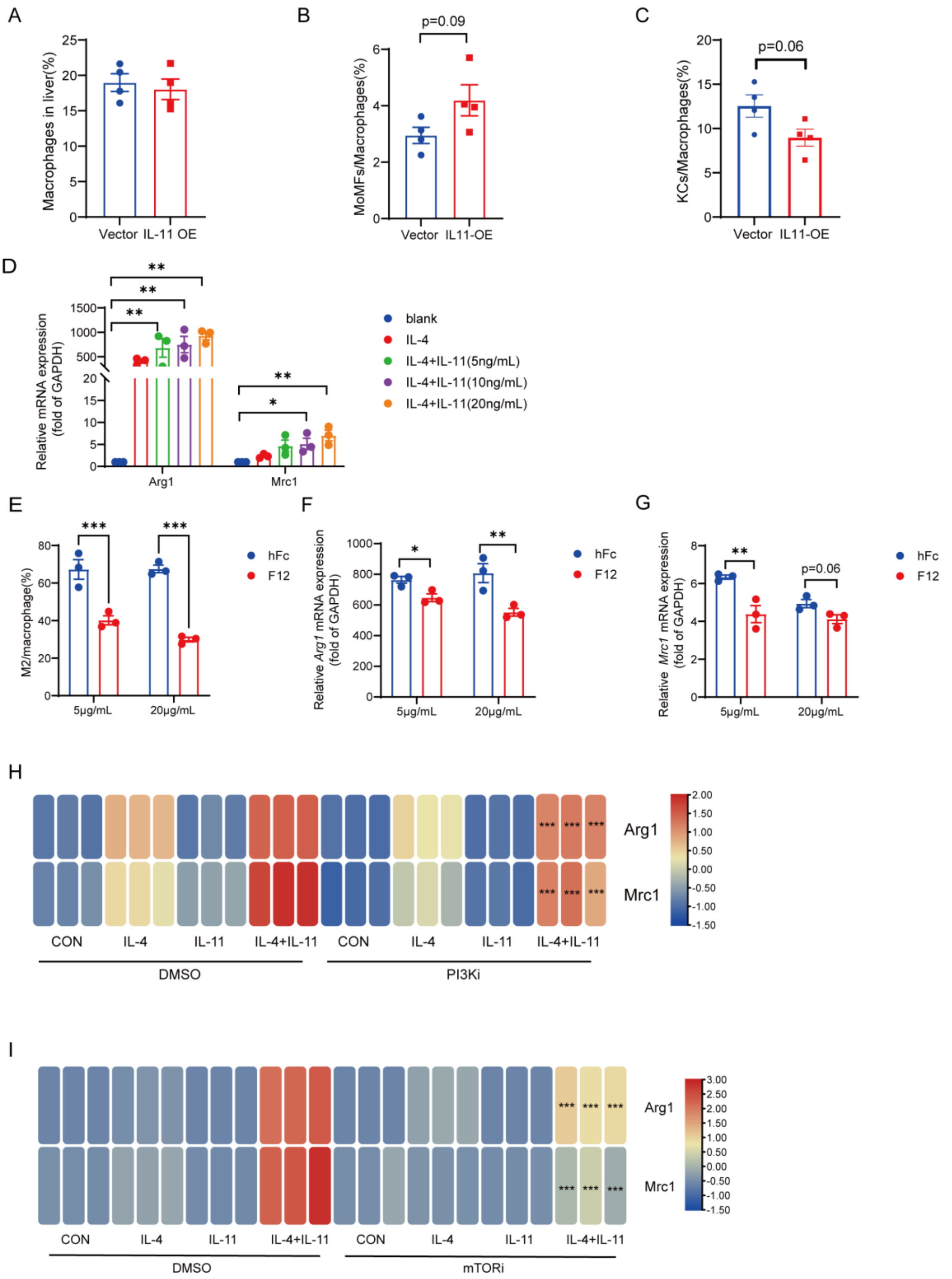

Supplementary Figure 4. The effect of IL-11 on macrophages polarization both *in vivo* and *in vitro*. Quantification of the whole macrophages (A), MoMFs (B) and KCs (C) of liver in IL-11 OE CCl_4 model. (D) The dose-dependent effect of IL-11 on M2 marker genes (Arg1 and Mrc1) of BMDMs. (E) The effect of F12 on IL-11-faciliated CD206+BMDMs quantified by flow cytometry. (F-G) The effect of F12 at different concentrations on M2 marker genes (Arg1 and Mrc1) of BMDMs. (H) The M2-like marker

genes(Arg1 and Mrc1) could be suppressed by PI3Ki (LY294002,10μM) compared with DMSO. (I) The M2-like marker genes (Arg1 and Mrc1) could be suppressed by mTORi (Rapamycin, 100nM) compared with DMSO. Data are presented as the mean ± SEM. n≥3, **p*<0.05, ***p*<0.01, ****p*<0.001 *vs.* Vector, blank, hFc or IL-11+IL-4 plus DMSO.

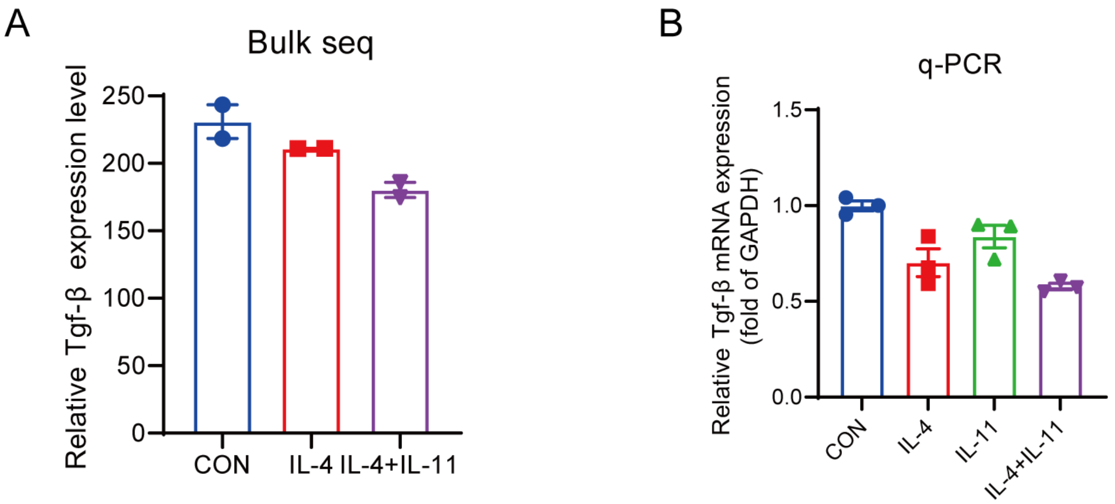

Supplementary Figure 5. Regulation of TGF-β stimulated by IL-11 was at translational level. (A) The transcriptional level of TGF-β in bulk sequencing result. (B) mRNA level of TGF-β was validated by q-PCR.

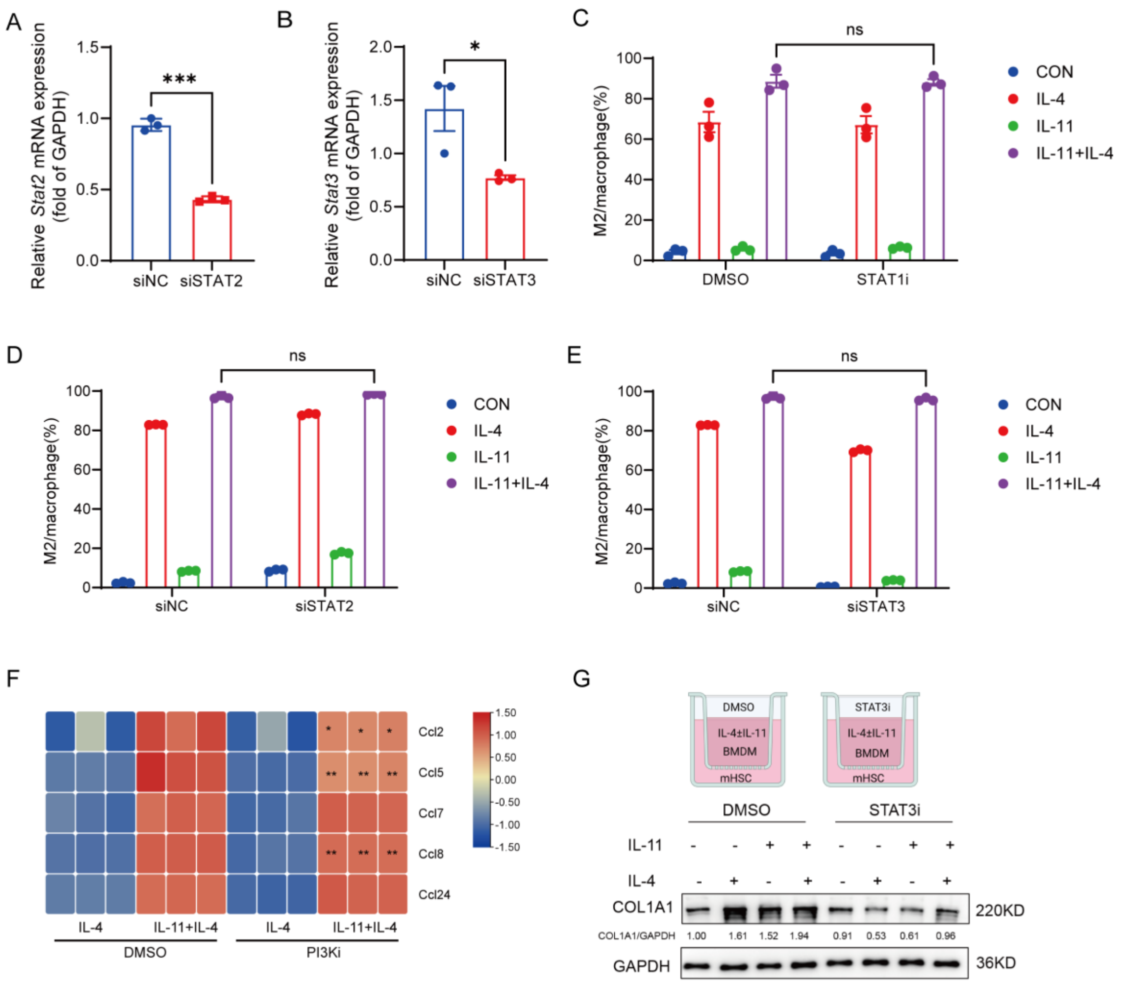

Supplementary Figure 6. STAT1, STAT2 and STAT3 involved in IL-11 induced M2-like polarization little, while PI3K marginally participated in IL-11 mediated CCL family members regulation. The knockdown efficiency of STAT2 siRNA (A) and STAT3 siRNA (B) in BMDM. (C) The effect of STAT1i (Fludarabine, 10μM) on IL-11±IL-4 induced M2 macrophages. The effect of STAT2 (D) or STAT3 (E) in in IL-11±IL-4 induced M2 macrophages analyze by flow cytometry. (F) Quantification of CCL family chemokines in BMDMs treated with 0.1%DMSO or PI3Ki (LY294002,10μM). (G) Western blot analysis of COL1A1 in mHSCs cocultured with IL-11(20ng/mL) and/or IL-4(40ng/mL)-treated with STAT3i (Stattic,5μM). Data are presented as the mean ± SEM. n≥3, *p<0.05, **p<0.01, ***p<0.001 *vs.* siNC, IL-11+IL-4 plus DMSO or IL-11+IL-4 plus siNC.

Table S1 List of primers for qRT-PCR

Gene	Species	Forward primer (5'->3')	Reverse primer (5'->3')
Col1a1	Mouse	GCTCCTCTTAGGGGCCACT	ATTGGGGACCCTTAGGCCAT
Col3a1	Mouse	CTGTAACATGGAAACTGGGGAAA	CCATAGCTGAACTGAAAACCACC
Acta2	Mouse	CCCAGACATCAGGGAGTAATGG	TCTATCGGATACTTCAGCGTCA
Il-11	Mouse	TGTTCTCCTAACCCGATCCCT	CAGGAAGCTGCAAAGATCCCA
Il-11ra	Mouse	TATTGGCGCTGGGAGGA	GCTGCTGCTCATCTTCTGC
Gp130	Mouse	CCGTGTGGTTACATCTACCCT	CGTGGTTCTGTTGATGACAGTG
Yap	Mouse	AGCAGCAGCAAATACAGCTGCAG	AGCATTTGCTGTGCTGGGATTGA
Ctgf	Mouse	AGAACTGTGTACGGAGCGTG	GTGCACCATCTTTGGCAGTG
Ankrd1	Mouse	GGAACAACGGAAAAGCGAGAA	GAAACCTCGGCACATCCACA
Cyr61	Mouse	TAAGGTCTGCGCTAAACAACTC	CAGATCCCTTTCAGAGCGGT
Arg1	Mouse	CTCCAAGCCAAAGTCCTTAGAG	AGGAGCTGTCATTAGGGACATC
Mrc1	Mouse	CATGAGGCTTCTCTTGCTTCTG	TTGCCGTCTGAACTGAGATGG
Spp1	Mouse	AGCAAGAAACTCTTCCAAGCAA	GTGAGATTCGTCAGATTCATCCG
Il-4ra	Mouse	TCTGCATCCCGTTGTTTTGC	GCACCTGTGCATCCTGAATG
Tgf-β	Mouse	CTCCCGTGGCTTCTAGTGC	GCCTTAGTTTGGACAGGATCTG
Ccl2	Mouse	TTAAAAACCTGGATCGGAACCAA	GCATTAGCTTCAGATTTACGGGT
Ccl5	Mouse	GCTGCTTTGCCTACCTCTCC	TCGAGTGACAAACACGACTGC
Ccl7	Mouse	GCTGCTTTCAGCATCCAAGTG	CCAGGGACACCGACTACTG
Ccl8	Mouse	TCTACGCAGTGCTTCTTTGCC	AAGGGGGATCTTCAGCTTTAGTA
Ccl24	Mouse	ATTCTGTGACCATCCCCTCAT	TGTATGTGCCTCTGAACCCAC
Stat2	Mouse	AATGGACGTTCGACAGCATT	TCTGAATATCCCGGCTGAAT
Gapdh	Mouse	AGGTCGGTGTGAACGGATTTG	GGGGTCGTTGATGGCAACA
YAP	Human	TAGCCCTGCGTAGCCAGTTA	TCATGCTTAGTCCACTGTCTGT
CTGF	Human	AAAAGTGCATCCGTACTCCCA	CCGTCGGTACATACTCCACAG
CYR61	Human	GGTCAAAGTTACCGGGCAGT	GGAGGCATCGAATCCCAGC
ANKRD1	Human	AGTAGAGGAACTGGTCACTGG	TGTTTCTCGCTTTTCCACTGTT
GAPDH	Human	GGAGCGAGATCCCTCCAAAAT	GGCTGTTGTCATACTTCTCATGG

Table S2 List of siRNA sequence

Gene	Species	Sequence(5'->3')
STAT2	Mouse	CCGGGAUAUUCAGACCUUUTT
STAT3	Mouse	CAUCAAUCCUGUGGUAUAATT
siNC	Mouse	UUCUCCGAACGUGUCACGUTT

Table S3 List of Antibodies

Immunoblotting		
Antibody	Catalog	Vendor
Collagen I	66761-1-Ig	Proteintech
αSMA	A17910	Abclonal
YAP	8418T	CST
p-YAP(S127)	4911	CST
COL3A1	A3795	Abclonal
GAPDH	60004-1-Ig	Proteintech
Flow cytometry		
Antibody	Catalog	Vendor
Fc block-anti-mouse CD16/32	101302	BioLegend
APC/Cy7 anti-mouse CD45.2	109824	BioLegend
PE/Cy7 anti-mouse F4/80	25-4801-82	eBioscience
BV421 anti-mouse CD11b	101235	Biolegend
APC anti-mouse CD86	560747	BD Biosciences
FITC anti-mouse CD206	141793	BioLegend
PE anti-mouse LAP(TGF-β1)	141306	BioLegend

References

1. Liu F, Sun C, Chen Y, Du F, Yang Y, Wu G. Indole-3-propionic Acid-aggravated CCl4-induced Liver Fibrosis via the TGF-β1/Smads Signaling Pathway. Journal of Clinical and Translational Hepatology 2021;9:917-930.
2. Ma N, Hou A, Pan X, Sun F, Xu X, Yu C, Lai R, et al. MiR-552-3p Regulates Multiple Fibrotic and Inflammatory genes Concurrently in Hepatic Stellate Cells Improving NASH-associated Phenotypes. International Journal of Biological Sciences 2023;19:3456-3471.
3. Rao J, Cheng F, Zhou H, Yang W, Qiu J, Yang C, Ni X, et al. Nogo-B is a key mediator of hepatic ischemia and reperfusion injury. Redox Biology 2020;37:101745.
4. Dong Y, Tang B-x, Wang Q, Zhou L-w, Li C, Zhang X, Sun D-d, et al. Discovery of a novel DDRs kinase inhibitor XBLJ-13 for the treatment of idiopathic pulmonary fibrosis. Acta Pharmacologica Sinica 2022;43:1769-1779.
5. Sasaki M, Miyakoshi M, Sato Y, Nakanuma Y. Modulation of the microenvironment by senescent biliary epithelial cells may be involved in the pathogenesis of primary biliary cirrhosis. Journal of Hepatology 2010;53:318-325.
